# Extra Virgin Olive Oil Secoiridoids Modulate the Metabolic Activity of Dacarbazine Pre-Treated and Treatment-Naive Melanoma Cells

**DOI:** 10.3390/molecules27103310

**Published:** 2022-05-21

**Authors:** Azra Kugić, Sanja Dabelić, Cvijeta Jakobušić Brala, Nina Dabelić, Monika Barbarić

**Affiliations:** 1 Faculty of Pharmacy and Biochemistry, University of Zagreb, A. Kovačića 1, 10 000 Zagreb, Croatia; akugic@student.pharma.hr (A.K.); sanja.dabelic@pharma.unizg.hr (S.D.); cjakobus@pharma.unizg.hr (C.J.B.); 2 Department of Oncology and Nuclear Medicine, University Hospital Center Sestre Milosrdnice, Referral Center for Melanoma of Ministry of Health of Republic of Croatia, Vinogradska 29, 10 000 Zagreb, Croatia; nina.dabelic@kbcsm.hr

**Keywords:** melanoma cells, olive oil, secoiridoids, oleocanthal, oleacein, dacarbazine, ^1^HNMR, HPLC-DAD

## Abstract

Nowadays, many individuals, whether healthy or diagnosed with disease, tend to expose themselves to various easily accessible natural products in hopes of benefiting their health and well-being. Mediterranean populations have traditionally used olive oil not only in nutrition but also in cosmetics, including skincare. In this study, the phenolic profile—composed of twelve compounds altogether, including the secoiridoids oleocanthal (OCAL) and oleacein (OCEIN)—of extra virgin olive oil (EVOO) from autochthonous cultivars from Croatia was determined using ^1^H qNMR spectroscopy and HPLC-DAD analysis, and its biological activity was investigated in melanoma cell lines. The EVOO with the highest OCEIN content had the strongest anti-cancer activity in A375 melanoma cells and the least toxic effect on the non-cancerous keratocyte cell line (HaCaT). On the other hand, pure OCAL was shown to be more effective and safer than pure OCEIN. Post-treatment with any of the EVOO phenolic extracts (EVOO-PEs) enhanced the anti-cancer effect of the anti-cancerous drug dacarbazine (DTIC) applied in pre-treatment, while they did not compromise the viability of non-cancerous cells. The metastatic melanoma A375M cell line was almost unresponsive to the EVOO-PEs themselves, as well as to pure OCEIN and OCAL. Our results demonstrate that olive oils and/or their compounds may have a potentially beneficial effect on melanoma treatment. However, their usage can be detrimental or futile, especially in healthy cells, due to inadequately applied concentrations/combinations or the presence of resistant cells.

## 1. Introduction

Nowadays, regardless of the unprecedented advances in modern medicine, people are increasingly turning to the enhanced usage of natural bioactive substances, available either over the counter or through domestic manufacture. With a long-standing tradition of olive oil production, Mediterranean populations are traditionally accustomed to using olive oil in nutrition and preparative cosmetics, including skincare. Depending on several factors, including the plant variety, cultivating conditions, and production process, the composition of olive oil can vary tremendously, making some particular oils or oil extracts more or less rich in biological compounds of known [1] or unknown impact on human health. Among these, of particular interest are undoubtedly phenolic compounds due to their numerous known biological effects ranging from antimicrobial and anti-inflammatory to cardioprotective and neuroprotective, and even anticancer [2]. According to epidemiological and pre-clinical studies, populations from Mediterranean countries who traditionally follow the Mediterranean diet, which has extra virgin olive oil (EVOO) as one of its main components, have a lower risk of chronic diseases related to various inflammations, including cancer [3]. The most important phenolic compounds in EVOO include phenol alcohols, hydroxytyrosol (HTyr), and tyrosol (Tyr) and their secoiridoid derivatives oleacein (OCEIN) and oleocanthal (OCAL), which can be found in olive oil in large quantities [4].

Turning to a healthy lifestyle and strengthening the immune system with the use of natural substances has become widely accepted, especially in the era of the COVID-19-pandemic, which affected many aspects of everyday life. During the course of the pandemic, there have been delays in cancer diagnostics, with the estimation that more cancers are now being diagnosed in advanced stages due to delayed patient reference and diagnosis and decreased healthcare accessibility. Many patients, including melanoma patients, have postponed cancer screening or diagnostics in fear of COVID-19 [5,6].

Although melanoma accounts for only about 1% of skin cancers, it is responsible for the vast majority of skin cancer deaths. It is estimated that in 2020, cutaneous melanoma accounted for 4% of all new cancer diagnoses in EU countries and 1.3% of all cancer-related deaths. This made it the sixth most frequently occurring cancer and one of the 20 most frequent causes of cancer death [7]. In one Mediterranean country, Croatia, in 2019, according to the Croatian National Cancer Registry, cutaneous melanoma was the eighth most common cancer in both males and females and accounted for 4% of new cancers in males and 3% of new cancers in females [8]. The melanoma treatment protocol depends on the cancer stage at the time of the diagnosis. In early-stage melanoma, surgical removal with adequate margins is curative. If regional lymph nodes are involved, surgical excision is followed by adjuvant systemic treatment, either targeted or immunotherapy. In distant metastatic disease, systemic therapy is indicated, i.e., checkpoint-inhibitor immunotherapy or targeted therapy (the latter in BRAF-mutated disease). The alkylating agent dacarbazine (DTIC) has been used in melanoma chemotherapy, either as monotherapy or as part of polychemotherapy protocols, since 1984 [9,10,11]. Nowadays, it is used upon disease progression in more effective treatment approaches as palliative therapy.

Melanoma cells’ response to polyphenols, including curcumin, quercetin, polyphenols derived from green tea, cruciferex, anthocyanins, ellagitanins, resveratrol, and others, has been investigated in various studies [12,13,14,15]. The anticancer activity of polyphenols is present primarily due to their high antioxidant capacity [16,17] and their ability to dispose of free radicals and modulate cellular signalling pathways and gene expression [18,19,20]. However, there are likely other mechanisms that are related to anti-tumour activity at the level of metastasis [21].

The effects of the following secoiridoids from olive oils on the inhibition of melanoma cell growth have been described in the literature: OCEIN [22,23], OCAL [24,25], and phenolic alcohol HTyr [23,26,27,28]. OCEIN was shown to have antitumor activity in melanoma cells by Carpi et al. [22], while other in vitro tests demonstrated its considerable effect on the growth of the human amelanotic melanoma C32 cell line [23]. Although the anti-inflammatory and anticancer characteristics of OCAL have been recognised [29,30], only a few studies have dealt with the effect of OCAL on human melanoma cells. In one in vitro study, OCAL demonstrated a remarkable and selective activity on A375 human melanoma cells in comparison to normal dermal fibroblasts and inhibited cancer signalling pathways [24]. OCAL was also found to reduce tumour growth in a subcutaneous xenograft model in vivo and inhibit the proliferation, invasion, migration, and angiogenesis of melanoma cells in vitro [25]. It was shown in another study that the anti-proliferative and pro-apoptotic potential of HTyr remarkably reduced the cell viability of melanoma cells, triggering apoptotic cell death [27]. Another study confirmed that HTyr showed significant anti-proliferative activity against melanoma cells at high concentrations, whereas cytoprotective activity was observed at lower concentrations [23]. This double effect agrees with the already known double effect of phenolic systems, which can act as either antioxidants or pro-oxidants, depending on concentration levels [31]. In experiments where human melanoma cells were used as model cells, HTyr prevented protein damage caused by longwave ultraviolet radiation [26] and affected metabolic and signalling pathways [28].

**Figure 1:**
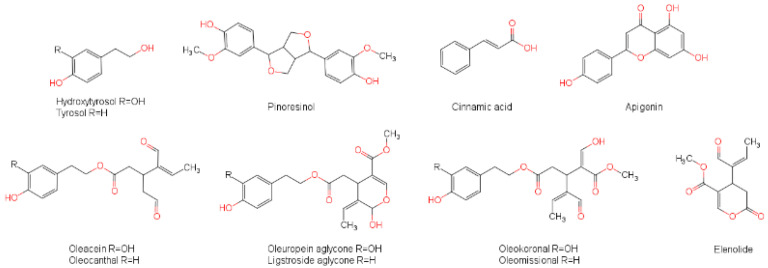
Structures of phenolic compounds/secoiridoids in studied EVOOs.

In light of the potential usage of olive oils as a natural product in the treatment of skin, which can contain early-stage or even metastatic melanoma cells potentially treated with DTIC, we conducted this study on the effects of EVOO polyphenolic extracts (EVOO-PEs) on melanoma cells, which to the best of our knowledge is first of its kind described in the literature. Both non-metastatic and metastatic melanoma cells and comparative non-tumorous keratinocytes were exposed to the various concentrations of EVOO-PEs, pure OCEIN and OCAL, and the anti-melanoma drug DTIC prior to treatment. To analyse the observed effects in the context of the EVOO phenolic profile, the contents of the bioactive compounds OCEIN, OCAL, and HTyr, along with nine other compounds (Figure 1), were determined using ^1^H qNMR spectroscopy and HPLC chromatography in three different olive oils.

## 2. Results and Discussion

### 2.1. EVOO’s Polyphenolic Profile

There are several methods for the quantification of EVOO polyphenols/secoiridoids; the most commonly used method is liquid chromatography coupled with UV-Vis/MS detection [32,33,34]. However, nuclear magnetic resonance (NMR) spectroscopy, as a powerful tool for the investigation of complex mixtures, has increasingly been applied for the characterization of olive oils in general [35,36,37] and particularly for the determination of olive oil polyphenols [38,39,40]. The ^1^H qNMR method developed by Karkoula et al. [41] has recently been used for the quantification of important bioactive EVOO secoiridoids, including OCEIN, OCAL, and a few others [42], as well in studies concerning their biological activities [43,44,45].

Here, the polyphenolic profiles of three different EVOOs from autochthonous Croatian cultivars—Bjelica, Žižolera, and Crnica—were determined. EVOO is well known as a valuable source of polyphenolic/secoiridoid compounds. This is important if we consider the beneficial effect of EVOO on human health, but we should also consider EVOO as a source of these valuable bioactive compounds for the investigation of their biological activities. There is great interest in the bioactive properties of compounds such as OCAL and OCEIN [46,47], and the number of studies is still rapidly increasing; however, there is no evidence regarding other compounds, such as oleokoronal, oleomissional, and *S*-(*E*)-elenolide, which are not commercially available as pure standards. We selected the EVOOs from different cultivars anticipating different polyphenolic profiles, which was necessary to analyse the relation between polyphenolic content and the effect of the EVOO-PEs on tumour cells. The EVOOs were characterized in terms of the content of total polyphenols, *o*-diphenols, and total flavonoids, and the content of the following individual polyphenolic compounds: the major secoiridoids OCAL and OCEIN, oleuropein aglycone, ligstroside aglycone, oleokoronal, oleomissional, *S*-(*E*)-elenolide, HTyr, Tyr, cinnamic acid, pinoresinol, and apigenin. Until now, Croatian olive oils have scarcely been characterized regarding their content of OCAL, OCEIN, oleuropein aglycone, ligstroside aglycone, oleokoronal, oleomissional, and *S*-(*E*)-elenolide. Bilušić et al. determined the content of OCAL, OCEIN, oleuropein aglycon, and ligtroside aglycon in olive oils from the Croatian cultivars Buhavica, Drobnica, Lastovka, Oblica, and Krvavica [48,49].

#### 2.1.1. Total Polyphenols, *o*-Diphenols, and Total Flavonoids

The content of total polyphenols (TPs), *o*-diphenols, and total flavonoids (TFs) was determined spectrophotometrically in EVOO-PEs following the procedures described in the literature [50,51,52]. EVOO-PEs were prepared using the optimized ultrasonic-assisted liquid-liquid extraction technique (US-LLE) with methanol and hexane [53]. The TP, *o*-diphenols, and TF values are summarized in Table 1. The TP content in the analysed EVOOs was around 400 mg/kg, placing them in the category of olive oils with an average TP content [54]. The TP content in the three EVOOs was fairly similar, with decreasing values in the following order: Bjelica, Žižolera, and Crnica. Although the EVOOs were similar regarding their TP content, there was a noticeable difference in *o*-diphenols content. This group of compounds is characterized by greater antioxidant activity in comparison to other phenolic compounds, a property related to the intramolecular stabilization of radicals formed in the course of antioxidative redox reactions; thus, *o*-diphenols are especially interesting in the evaluation of the bioactive properties of EVOO polyphenols. The highest content was determined in Žižolera, followed by Crnica and Bjelica. Interestingly, the fraction of *o*-diphenols in the total polyphenol content was the lowest in Bjelica (29%), which had the largest TP content, while the upper fraction value was obtained for Žižolera (57%). In addition, there was a significant difference in TF content among analysed oils, with decreasing values in the following order: Žižolera, Bjelica, and Crnica. Altogether, the three analysed oils had approximately the same amount of TPs, while Žižolera was the richest in *o*-diphenols and flavonoids.

#### 2.1.2. Secoiridoids Determined by NMR Spectroscopy

**Figure 2:**
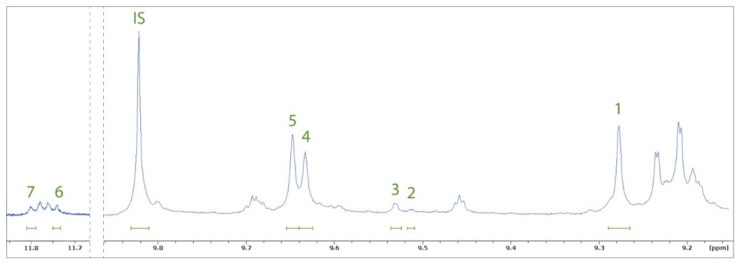
^1^H NMR spectrum of the aldehydic region of EVOO-PEs from the Crnica cultivar. 1—(*S*)-(*E*)-elenolide; 2—ligstroside aglycone; 3—oleuropein aglycone; 4—oleocanthal; 5—oleacein; 6—oleokoronal; 7—oleomissional; IS—internal standard (syringaldehyde).

The concentrations of the secoiridoid polyphenolic compounds OCEIN, OCAL, oleuropein aglycone, ligstroside aglycone, oleokoronal, oleomissional, and *S*-(*E*)-elenolide were determined using ^1^H qNMR spectroscopy following the method introduced by Karkoula et al. [41,55,56]. Prior to NMR analysis, polyphenolic compounds were extracted from the EVOOs using acetonitrile and cyclohexane. It is worth noting that this extraction procedure is much easier and faster than the extraction used for sample preparation before HPLC analysis (described in Section 2.1.3). The measurements were performed using an NMR instrument operating at 600 MHz to avoid problems with peak overlaps. In the aldehydic proton region in the ^1^H NMR spectrum of acetonitrile EVOO-PEs, a set of peaks corresponding to the analysed compounds between 9.25 ppm and 11.85 ppm were used for their quantitative determination (Figure 2 and Figure S1). OCEIN, OCAL, oleuropein aglycone, and ligstroside aglycone were quantified by integrating their aldehydic proton signals at 9.65 ppm, 9.63 ppm, 9.53 ppm, and 9.51 ppm, respectively. Oleokoronal and oleomissional were quantified using the integration of their enolic proton signals at 11.74 and 11.80 ppm, respectively, while (*S*)-(*E*)-elenolide was quantified by integrating its proton signal at 9.28 ppm. The calibration curves of OCEIN and OCAL are presented in Figure S2, while the calibration curves of other analysed compounds were obtained from the literature [42,57]. The OCEIN and OCAL proton signals at 9.65 ppm and 9.63 ppm were integrated since they have been proven to be more reliable for the purpose of quantitative determination than their doublet signals found at 9.21 ppm and 9.23 ppm. It has been reported that the latter signals, especially in the case of OCEIN, are known to overlap with other nearby signals in the spectrum [41,55]. The determined concentrations of phenolic compounds are presented in Table 2.

Among the determined compounds, OCAL and OCEIN are particularly interesting considering their bioactive properties. OCAL is mainly recognized as a compound with neuroprotective activity in Alzheimer’s disease [58], although it also has anti-inflammatory and anticancer activities [59,60,61], while OCEIN is widely known for its antioxidative, anti-inflammatory, anti-atherosclerotic, and neuroprotective activities [62,63,64]. The concentration of OCAL in the analysed EVOOs ranged from 125 to 215 mg/kg EVOO, and the concentration of OCEIN was in the range of 125–330 mg/kg EVOO. For illustration, according to Karkoula et al. [41], the concentrations of OCAL and OCEIN in the top ten highest Greek EVOOs were in the range of 180–350 mg/kg EVOO and 100–290 mg/kg EVOO, respectively. The greatest concentration of OCAL was found in EVOO from the Bjelica cultivar (215 mg/kg), with the lowest concentration of OCEIN (125 mg/kg), while Crnica was the richest in OCEIN. Interestingly, the concentration of OCEIN in Žižolera and Crnica was about twice as high as the concentration of OCAL, while their ratio was approximately reciprocal in Bjelica, i.e., around 0.5. Oleuropein aglycone and ligstroside aglycone are bioactive compounds as well and have been previously determined to have anticancer activity, mainly in the case of breast cancer [47,65,66]. Oleuropein aglycone has also been shown to have neuroprotective activity against Alzheimer’s [67] and Parkinson’s disease [68]. In this study, the three analysed oils differed greatly in oleuropein aglycone and ligtroside aglycone content. The greatest concentration of oleuropein aglycone was found in Žižolera (215 mg/kg), which was tenfold higher than the lowest content found in Crnica (19 mg/kg). The concentration of ligstroside aglycone was much lower than the concentration of oleuropein aglycone in all analysed oils, ranging from 5 to 50 mg/kg, while the greatest concentration was present in Bjelica. Furthermore, the concentrations of oleokoronal, oleomissional, and *S*-(*E*)-elenolide were determined according to the method recently proposed by Diamantakos et al. [42,57]. Until now, there has been no reported evidence regarding the biological activity of these compounds. However, when considering the similarities between their structures and other secoiridoids, it could be expected that they would also possess some activities. The concentration of oleokoronal was approximately the same in all three oils, around 100 mg/kg, with the highest value found in Bjelica (150 mg/kg), while the concentration of oleomissional was the lowest in this oil. A notably high concentration of *S*-(*E*)-elenolide was measured in Žižolera (1050 mg/kg). Rigakou et al. [57] analysed 2120 olive oil samples and concluded that the *S*-(*E*)-elenolide concentration in around 40% of the samples had a value in the range of 1–175 mg/kg, with just 5% of samples in the highest range of 611–2821 mg/kg.

#### 2.1.3. Phenolic Compounds Determined by HPLC Chromatography

Five phenolic compounds—the simple polyphenols HTyr, tyrosol (Tyr), and cinnamic acid and the flavonoids apigenin and lignan pinoresinol—were determined by HPLC chromatography using a DAD detector following the previously described procedure [69,70] (Figure S3). Prior to HPLC analysis, polyphenols were extracted from olive oil by US-LLE using methanol and hexane [53]. HTyr [71,72] and Tyr [73] are phenolic compounds specific to olive oil, while cinnamic acid [74], apigenin [75], and pinoresinol [76] are widespread and found in a great number of plants. All of these compounds are well known for their bioactive properties, such as antioxidant and antitumor activities, in addition to many others [77,78]. All analysed compounds were present in low concentrations, below 10 mg/kg. Similar values were previously obtained for other Croatian olive oils [69,79].

### 2.2. Biological Effects of EVOO-PEs, DTIC, OCEIN, and OCAL on the Metabolic Activity of A375, A375M, and HaCaT Cells

**Figure 3:**
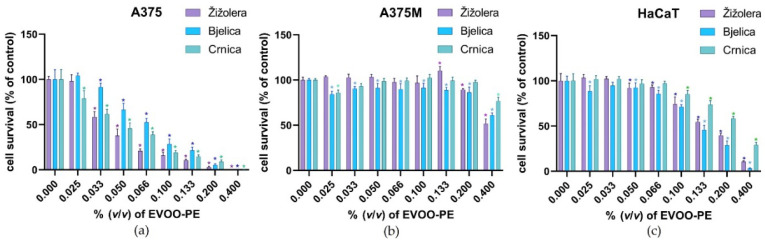
Effect of various concentrations of EVOO-PEs derived from different cultivars on cellular metabolic activity. Three cell lines, the (**a**) A375 melanoma cell line, (**b**) the A375M metastatic melanoma cell line, and (**c**) HaCaT immortalized keratinocytes, were exposed for 48 h to various concentrations of EVOO-PEs derived from different cultivars (Žižolera, Bjelica, and Crnica). Metabolic activity was measured using an MTS assay. The results of the six experiments are expressed as a mean percentage of cell viability compared to non-treated cells ± SD. Statistically significant differences (*p* < 0.05) compared to non-treated cells are marked with an asterisk.

Biological activity upon treatment with EVOO-PEs, DTIC, OCEIN, and OCAL was evaluated using an MTS assay in in vitro experimental models, including the A375 and A375M cancer cell lines and HaCaT immortalized cell line. The preliminary experiments we performed using several initial seeding densities and incubation times (24, 48, and 72 h; data not shown) revealed that the most informative results could be obtained with a seeding density of 2 × 10^3^ cells/well for A375 and HaCaT cells and 7 × 10^3^ cells/well for A375M cells, as well as exposure to test compounds for 48 h. Due to the fast cell growth rate, a longer incubation period or denser initial seeding caused the overgrowth of untreated cells. In comparison, a shorter incubation period or lower initial density caused the effect of the test substances to be more difficult to detect. Therefore, the experiments were performed within a 48-hour timeframe. In addition, to avoid the misinterpretation of the results, we checked whether the vehicle, which was DMSO for the test substances, had any effect on metabolic activity by exposing each cell type to 0.2 and 0.4% DMSO, i.e., to the highest final DMSO concentrations achieved with the highest final concentrations of test substances. The results showed no difference in the biological activity of cells exposed to a complete medium compared to those exposed to 0.2 and 0.4% DMSO (Figure S4). Accordingly, the effects of the test substances were determined as relative biological activities (in terms of cell viability, i.e., cell survival) compared to the untreated cells (controls). The cells were treated with a series of dilutions made by diluting full strength (100%) EVOO-PE to concentrations of 0.025–0.400 % (*v*/*v*) of EVOO-PE in the medium. As shown in Figure 3, all EVOO-PEs significantly inhibited A375 cells in a dose-dependent manner, showing a classical dose–response curve with complete inhibition achieved with the highest applied concentration (0.4%) of all EVOO-PEs.

While the EVOO-PEs of Žižolera and Crnica had almost the same dose–response curve, Bjelica was determined to be slightly less toxic at the same concentration. To the contrary, Crnica was shown to be less toxic to HaCaT cells than Žižolera and Bjelica, which had a similar effect on the HaCaT cells with a shift towards higher concentrations (i.e., IC_50_ was much higher); Bjelica was shown to have a slightly stronger toxic effect (Figure 3, Table 3).

Regarding its composition profile, among the analysed oils, EVOO Crnica was characterized by the highest amount of oleacein, while its contents of oleuropein aglycone and hydroxytyrosol were fairly low. Additionally, when comparing EVOO Crnica to the large set of oils (2120 samples) profiled by Rigakou et al. [57], in which just 5% of samples had values in the highest range (611–2821) mg/kg of *S*-(*E*)-elenolide, it was concluded that the 500 mg/kg detected in Crnica was a relatively high value. Žižolera’s higher toxicity in HaCaT cells and similar activity in A375 cells (in comparison with Crnica) is likely related to its higher content of TP, *o*-diphenols, and TF, and is in agreement with the possible pro-oxidative activity of polyphenols at higher concentrations suggested by Stevenson et al. [31]. Only the highest applied concentration of any of the EVOO-PEs caused a biologically significant decrease in the metabolic activity of A375M cells, which prevented the reliable determination of IC_50_ values, though a modest reduction in metabolic activity (up to 15%) was visible even using smaller concentrations of Bjelica.

OCEIN and OCAL failed to provoke any effect on A375M cells (Figure S5b, Table 3). OCAL showed a similar dose–response effect on A375 and HaCaT cells compared to the investigated EVOO-PEs, meaning that higher concentrations were needed to decrease metabolic activity in HaCaT than in A375. OCEIN also showed a dose–response effect; however, similar concentrations provoked the same effect on both A375 and HaCaT cell lines, meaning that the positive toxic effect on tumour cells is unfortunately accompanied by a negative toxic effect on non-tumour cells. Taken together, it was observed that OCAL was more potent in tumour A375 cells and less toxic to non-tumour HaCaT cells than OCEIN (Figure S5, Table 3), while both were ineffective in metastatic melanoma cells.

Until today, the influence of EVOO-PEs on melanoma cells has not been described in the literature, while data on the impact of its two components, OCEIN and OCAL, are scarce. The IC_50_ values of OCAL determined by both Fogli et al. [24] and Gu et al. [25] in A375 cells were slightly smaller than those determined by us (13.6 µM and ~20 µM vs. 67 µM, respectively), which can be explained by the fact that those two research groups used homemade isolated OCAL, which, though purified to 95%, might have contained some other substances from that particular oil, which contributed to the toxicity in A375 cells. It is worth mentioning that Gu et al. [25] detected exactly the same impact of their highest (60 µM) applied concentration of OCAL as us (reduction to ~80% viability compared to control). Thus far, the known effects of OCEIN on melanoma cells vary dramatically from cytotoxic to cytoprotective. Our findings are in agreement with the results of Carpi et al. [22], who also detected an inhibitory effect of OCEIN within the same concentration range on 501Mel melanoma cells as we did in A375 melanoma cells (IC_50_ being 82 µM vs. 112 µM, respectively). To the contrary, de Carvalho et al. [23] found OCEIN to be an inducer of cell proliferation in C32 amelanotic melanoma cells. Taking into consideration that both cell lines, C32 and A375, share amelanotic properties, these differences cannot be explained by the cell type itself and need further investigation.

Analysing the effect of EVOO-PEs on cell viability in the context of their composition profile regarding OCEIN and OCAL, it was noticed that all EVOO-PEs, at the concentration that caused particular inhibition (e.g., IC_60_), contained either OCEIN or OCAL in tens of times lower concentrations than needed for pure substances themselves to achieve the same inhibitory effect. Although this was not particularly surprising and unexpected because of the anticipated influence of other EVOO components, which might have additional or even synergistic effects, it is interesting to note the proportions for specific EVOOs. At IC_60_ values for A375 cells, the phenolic extracts of Žižolera, Bjelica, and Crnica contained 0.61 μM, 0.35 μM, and 0.92 μM of OCEIN, respectively, and 0.36 μM, 0.63 μM, and 0.46 μM of OCAL respectively, while pure OCEIN had IC_60_ at 101.052 μM and OCAL at 58.488 μM.

DTIC, applied in concentrations up to 1000 µM, also showed a dose-dependent effect; however, the highest concentration of DTIC did not completely inhibit any type of cells (Figure S6). Therefore, these IC_60_ and IC_50_ values should be interpreted with caution. Yet, unlike the other test compounds, A375M cells responded even to a slightly lower dose of DTIC. The toxic effect was most pronounced on A375 cells, followed by A375M and then HaCaT cells. The IC_60_ and IC_50_ values calculated for all applied EVOO-PEs and substances are summarized in Table 3.

### 2.3. Post-Treatment Biological Effects of EVOO-PEs on the Metabolic Activity after Pre-Incubation with DTIC

**Figure 4:**
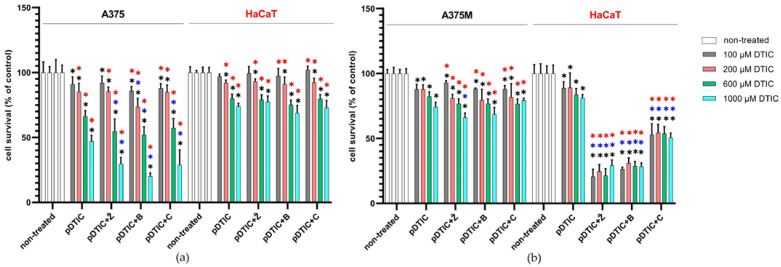
Effect of EVOO-PEs derived from three different cultivars (Žižolera, Bjelica, and Crnica) on cellular metabolic activity after pre-treatment with DTIC. Cells were pre-treated with various concentrations of DTIC (100, 200, 600, or 1000 µM) for 6 h and afterwards exposed for 24 h to EVOO-PEs in concentrations corresponding to the IC_60_ values for (**a**) the A375 melanoma cell line and (**b**) the A375M metastatic melanoma cell line. In both cases, comparative non-cancerous HaCaT cells were exposed to the same concentrations of EVOO-PEs as applied to A375 and A375M cells. Metabolic activity was measured using an MTS assay. The results of the six experiments are expressed as mean percentage of cell viability compared to non-treated cells ± SD. Statistically significant differences (*p* < 0.05) are marked as follows: black asterisk—compared to the non-treated cells; blue asterisk—compared to the cells pre-treated with DTIC; red asterisk—difference between melanoma and HaCaT cells. Legends: pDTIC—pre-treatment with DTIC; pDTIC + Ž, pDTIC + B, pDTIC + C—pre-treatment with DTIC followed by exposure to EVOO-PEs derived from three different cultivars, Žižolera, Bjelica, and Crnica, respectively.

A comparison of the dose–response curves of A375 and HaCaT cells for all tested EVOO-PEs showed that at concentrations that caused a 40% inhibition of the metabolic activity of A375 cells (i.e., IC_60_), there was no statistically and/or biologically significant reduction in the metabolic activity of non-tumorous HaCaT cells (Figure 3, Table 3). Therefore, those concentrations (0.036% of EVOO-PE for Žižolera, 0.057% of EVOO-PE, for Bjelica and 0.037% of EVOO-PE for Crnica) were selected to conduct experiments in which cells were pre-incubated with various doses of DTIC (100, 200, 600, and 1000 µM) for 6 h and then exposed to EVOO-PE for 24 h. Similarly, A375M and HaCaT cells were exposed to EVOO-PE IC_60_ for that type of the cancerous cells (0.355% Žižolera and 0.401% Bjelica) and to the highest tested concentration (0.400%) of Crnica (for which a reduction to 75% of the initial value of A375M was achieved). Treatment after smaller doses of DTIC (100 µM and 200 µM) did not affect the metabolic activity of either A375 or HaCaT cells, except to a small extent (~10%) in the case of Bjelica in A375 cells. After the administration of higher doses of DTIC (600 µM and 1000 µM), the toxic effect on A375 was enhanced under the influence of all tested EVOO-PEs, causing an additional reduction in metabolic activity of approximately 10 and 20%, respectively, while there was no change in the metabolic activity of HaCaT cells (Figure 4a).

To the contrary, post-treatment with any EVOO-PE did not affect the metabolic activity of A375M cells; only a very modest reduction (by 5%) was observed with a combination of 1000 µM DTIC and EVOO-PE Žižolera. Yet, at the same time, all EVOO-PEs significantly reduced the metabolic activity of HaCaT cells at all used DTIC concentrations. Crnica had the lowest toxic effect, causing a decrease in metabolic activity by approximately 30%, while Žižolera and Bjelica had a stronger effect, both reducing metabolic activity by more than 50% (Figure 4b). The observed effect could be a consequence of the pro-oxidative activity of TP and *o*-diphenols when they are present at higher concentrations [31].

**Figure 5:**
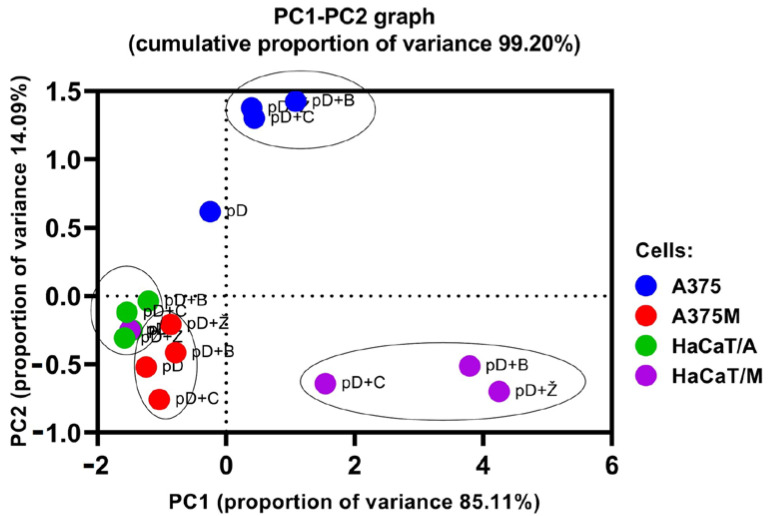
Principal component analysis (PCA) score plot. Principal component analysis (PCA) was performed on the data obtained from the measurement of cellular metabolic activity after 6 h pre-treatment with DTIC followed by the 24 h treatment of A375 and A375M cells with IC_60_ concentrations of EVOO-PEs derived from three different cultivars (Žižolera, Bjelica, and Crnica). Comparative non-cancerous HaCaT cells were exposed to the same concentrations of EVOO-PEs as applied to A375 and A375M cells. Legends: pD—pre-treatment with DTIC; pD + Ž, pD + B, pD + C—pre-treatment with DTIC followed by exposure to EVOO-PEs derived from three different cultivars, Žižolera, Bjelica, Crnica, respectively; HaCaT/A and HaCaT/M—HaCaT cells exposed after DTIC pre-treatment to the same concentrations of EVOO-PEs as applied to A375 and A375M cells, respectively.

A principal component analysis (PCA) was carried out on the biological activity data obtained in the experiments with DTIC pre-treatment followed by EVOO-PE exposure. The PCA showed that the first two principal components explained 99.01% of the total variance (Figure 5). The first accounted for 84.64% and the second for 14.37%.

The biplot of PC1 and PC2 shows grouping in four distinct groups, while two-and-two share important characteristics (Figure 5). All red dots (A375M) and green dots (HaCaT/A) are grouped together, regardless of whether or not pre-treatment with pDTIC was followed by post-treatment with EVOO-PE. This confirms that EVOO-PE post-treatment did not have a significant impact on A375M cells, nor on HaCaT cells which were used as comparative cells in the A375 experiments (i.e., cells designated as HaCaT/A). To the contrary, the two other groups (A375 and HaCaT/M) were separated from each other and from the corresponding cells that were only pre-treated with DTIC, indicating the different effect of EVOO post-treatment compared to pre-treatment with DTIC; this effect was of different intensity and cell-type dependent.

The molecular mechanisms underlying the observed effects are still to be elucidated, and we can only speculate whether they are the same as those suggested in the investigations performed by other researchers on different cell-types [80], using various oils or oil substances, and/or varying experimental parameters, etc. Based on these studies, it is reasonable to assume that the response of melanoma cells to EVOOs includes, but is not limited to, signalling pathways, such as protein-serine/threonine kinases [24,28,80], or target genes, such as Bcl-2 [24], B-Raf [80], Bcl-xL, MMP-2, MMP-9, VEGF [25], p53, and γH2AX [27]. Additionally, it would be interesting to explore the role of cellular importing and exporting mechanisms and their contribution to the overall effect of tested substances.

Altogether, the results of this study indicate that EVOOs have the potential to be used as auxiliary substances in the treatment of melanoma and that they may enhance the positive effects of conventional therapies. However, metastatic cells bearing numerous mutations remain to be an unconquerable fortress in our constant battle against melanoma, whether we are using chemotherapeutics, immunotherapy, targeted therapy, or natural bioactive compounds.

## 3. Materials and Methods

### 3.1. Reagents and Standards

Syringaldehyde, deuterated chloroform (D, 99.8%), sodium molybdate dihydrate, hydroxytyrosol, apigenin, gallic acid, catechin, and pinoresinol were purchased from Sigma-Aldrich (Steinheim, Germany). Glacial acetic acid, acetonitrile, cyclohexane, aluminium chloride, sodium carbonate anhydrous, sodium nitrite, and methanol (HPLC grade) were obtained from Merck (Darmstadt, Germany). Folin–Ciocalteu’s reagent, tyrosol, and cinnamic acid were bought from Fluka Chemie (Buchs, Switzerland), while oleacein and oleocanthal were obtained from PhytoLab GmbH (Vestenbergsgreuth, Germany) and dacarbazine from Selleck Chemicals (München, Germany). Sodium hydroxide and *n*-hexane were purchased from Kemika (Zagreb, Croatia). Components for cell culture maintenance, including RPMI 1640, FBS, trypsin-EDTA, phosphate-buffered saline (PBS; Ca^2+^ and Mg^2+^ free), penicillin, and streptomycin, were from Lonza (Basel, Switzerland) and the CellTiter 96^®^AQueous One Solution Cell Proliferation Assay was from Promega (Madison, WI, USA).

### 3.2. Olive Oil Samples

This study included three different monovarietal EVOO samples produced from autochthonous olive cultivars cultivated on the Istrian peninsula in Croatia. The olive oil samples produced solely by mechanical means were obtained from two manufacturers: EVOOs from the Žižolera and Bjelica cultivars were produced by Oleum Maris d.o.o. (Vodnjan, Croatia), while the EVOO from the Crnica cultivar (Vodnjanska Crnica) was produced by Olea B.B. d.o.o. (Rabac, Croatia). A commercially obtained refined olive oil was used to prepare calibration curves for OCAL and OCEIN. The olive oil samples were stored in dark glass bottles at 4 °C and kept out of light.

### 3.3. Extraction of Polyphenolic Compounds for Spectrophotometric and HPLC-DAD Analysis

Polyphenolic compounds were extracted from EVOOs using the US-LLE technique, following the procedure described in the literature with some modifications [53]. A total of 20.0 g of EVOO sample was dissolved in 10 mL of *n*-hexane; 15 mL of methanol was added, and the mixture was sonicated (3 × 10 min) using an ultrasonic bath (Elma Transonic T570 HF = 320 W, Germany). Homogenates obtained from three extraction phases were separated in two steps using a centrifuge for 15 min at 4000 rpm (Hettich centrifuge D-78532, Tuttlingen, Germany). The obtained methanol phase containing polyphenolic compounds was degreased with *n*-hexane and concentrated using a rotary evaporator at 38 °C (Büchi Heating Bath B-490, Büchi Labortechnik AG, Flawil, Switzerland) till dryness. The dry extract was used immediately for spectrophotometric and HPLC-DAD analysis. EVOO-PE stock solution was prepared by dissolving the dry extract in methanol.

### 3.4. Polyphenolic Compounds Determined by Spectrophotometric Analysis

#### 3.4.1. Total Polyphenols Analysis

The concentration of total polyphenols (TPs) in EVOO was determined spectrophotometrically with Folin–Ciocalteu (FC) reagent [50]. A total of 0.1 mL of EVOO-PE solution, 5 mL of water, and 0.25 mL FC reagent were added to a 10 mL volumetric flask. A 1.5 mL aliquot of saturated (20%) sodium carbonate solution was added to the reaction mixture after 3 min and diluted with water to 10 mL. After 30 min, the absorbance of this solution was measured at 725 nm (UV-VIS, Hewlett Packard 8453, Böblingen, Germany). The calibration curve was prepared with gallic acid (Table S1), and the concentration of TP in EVOO was expressed in mg gallic acid equivalent (GAE)/kg of EVOO.

#### 3.4.2. *o*-Diphenols Analysis

The concentration of *o*-diphenols in EVOO was determined spectrophotometrically with sodium molybdate [51]. A 5% sodium molybdate solution was prepared by dissolving sodium molybdate in a methanol:water (1:1 *v*/*v*) solvent mixture. Diluted extract solutions (D) were prepared by mixing 0.50 mL of the EVOO-PE stock solution with the corresponding volume of the methanol:water solvent (1:1 *v*/*v*). A 0.5 mL aliquot of 5% sodium molybdate solution was added to 2 mL of D solution, the solution was kept in the dark for 15 min, and the absorbance was measured at 350 nm. The calibration curve was prepared with gallic acid (Table S1), and the concentration of *o*-diphenols in EVOO was expressed in mg gallic acid equivalent (GAE)/kg of EVOO.

#### 3.4.3. Total Flavonoids Analysis

The concentration of total flavonoids (TFs) in EVOO was determined spectrophotometrically according to the previously described procedure [52]. A total of 1.00 mL of appropriately diluted EVOO-PE, 4 mL of water, 0.3 mL of 5% solution of sodium nitrite, and 0.3 mL of 10% solution of aluminium chloride were added to a 10 mL volumetric flask. The solution was kept in the dark for 5 min, and then 2 mL of 1 mol/L sodium hydroxide solution was added and it was filled with water. The absorbance of this solution was measured at 510 nm. The calibration curve was prepared with catechin (Table S1), and the concentration of total flavonoids in EVOO was expressed in mg catechin equivalent (CE)/kg of EVOO.

### 3.5. Secoiridoids Determined by NMR Spectroscopy

#### 3.5.1. Extraction of Phenolic Compounds/Secoiridoids for ^1^H NMR Analysis

The extraction process was carried out by mixing 5.00 g of olive oil with 25 mL of acetonitrile and 20 mL of cyclohexane, according to the procedure previously described by Karkoula et al. [41] with some modifications. The oil and extraction solvent mixture was homogenized using a vortex mixer for one minute and centrifuged for 5 min at 4000 rpm (Hettich centrifuge D-78532, Tuttlingen, Germany). The acetonitrile phase was separated and mixed with 1.0 mL of 0.5 mg/mL syringaldehyde solution prepared in acetonitrile, to be used as an internal standard. The solvent was removed using a rotary evaporator (Büchi Labortechnik AG, Flawil, Switzerland) at a temperature no greater than 33 °C. The dry residue was kept at −8 °C prior to NMR analysis. Throughout the entire procedure, the sample was minimally exposed to light and oxygen. Three dried phenolic extracts were obtained from each olive oil sample. Two phenolic extracts were used for ^1^H NMR analysis, while the third was dissolved in 2 mL of DMSO for biological activity tests.

#### 3.5.2. ^1^H qNMR Analysis

Dry polyphenolic extracts were dissolved in 750 μL of *d*-chloroform, and a volume of 550 μL of the solution was transferred to a 5 mm NMR tube. ^1^H NMR spectra were recorded at a frequency of 600.130 MHz (Bruker Avance 600) at 25 °C, while 64 scans were collected into 32 K data points over a spectral width of 0–20 ppm (12,000 Hz). A 5.0 s relaxation delay and an acquisition time of 1.36 s were used. The resulting ^1^H NMR spectra were used for the quantitative determination of the secoiridoid phenolic compounds OCAL, OCEIN, oleuropein aglycone, ligstroside aglycone, oleokoronal and oleomissional, and (*S*)-(*E*)-elenolide. The spectra were processed using the TopSpin program (TopSpin 4.0.7., Bruker, Billerica, MA, USA). The peaks of interest were manually integrated.

#### 3.5.3. ^1^H NMR Calibration Curves and Quantitative Determination

OCAL and OCEIN calibration curves were prepared by diluting specific volumes of stock standard solutions in 5.0 g of a commercially obtained refined olive oil (Table S2). The refined olive oil was used as a blank since it was determined that it did not contain OCAL or OCEIN in detectable quantities, and there were no interferences in the spectra overlapping the peaks of interest. Stock standard solutions of OCAL and OCEIN (0.5 mg/mL) were prepared in acetonitrile and stored at −8 °C. Their stability was controlled spectrophotometrically. A number of oil samples at seven concentration levels were prepared with concentrations of OCAL and OCEIN ranging from 10.0 to 350 mg/kg of oil. The extractions of phenolic compounds from these olive oil samples and their ^1^H NMR analyses were carried out as described in previous sections. The quantitative determination was based on the ratio of OCAL and OCEIN aldehydic proton signal surfaces at 9.63 ppm and 9.65 ppm, respectively, to the internal standard aldehydic proton signal surface at 9.82 ppm. Oleuropein aglycone and ligstroside aglycone, oleokoronal, oleomissional, and (*S*)-(*E*)-elenolide were quantified by integrating proton signals at 9.50 ppm, 9.48 ppm, 11.74 ppm, 11.80 ppm, and 9.27 ppm, respectively, and using the calibration curves supplied by Diamantakos et al. and Rigakou et al. [42,57].

### 3.6. Phenolic Compounds Determined by HPLC Chromatography

#### HPLC-DAD Analysis

HPLC-DAD analysis was performed according to the previously described procedure [69,70]. A Perkin Elmer Series 200 system (PerkinElmer, Waltham, MA, USA) with diode array detector was used, equipped with a C18 Restek column (5 μm, 250 × 4.0 mm). The gradient change of a mobile phase consisting of two components was used (A: 2% acetic acid and B: methanol) with an increasing percentage of B as follows: 5% B for 2 min; going to 25% B till 10 min; 40% B till 20 min; 50% B till 30 min; and 100% B till the end of the run at 45 min. Between two runs, the column was washed for 15 min with methanol. The flow rate was 1 mL/min and the injection volume was 25 μL at 25 °C. The absorbance was detected at 278 nm. The retention times of the analysed compounds were compared with those of standards (min): hydroxytyrosol (12.20), tyrosol (15.66), cinnamic acid (30.20), pinoresinol (33.41), and apigenin (42.65). The concentrations were calculated using calibration curves (Table S3).

### 3.7. Cell Culture and Biological Activity

Human melanoma cell lines A375 [81] and A375M [82] were kindly given to us by Neda Slade, PhD, from the Division of Molecular Medicine, Ruđer Bošković Institute, Zagreb, Croatia, while HaCaT [83], a spontaneously immortalized cell line of keratinocytes, was a gift from Professor Jasmina Lovrić, University of Zagreb, Faculty of Pharmacy and Biochemistry, Zagreb, Croatia. Originally, the melanoma cell lines were purchased from the American Type Culture Collection (Manassas, VA, USA) and HaCaT from Cell Line Services GmbH (Eppelheim, Germany).

Cells were grown in complete cell culture media, consisting of RPMI 1640, 2 mmol/L glutamine, 10% heat-inactivated foetal bovine serum (FBS), penicillin (100 IU/mL; 1 IU 67.7 µg/mL), and streptomycin (100 µg/mL). Cultures were maintained in a moisturized atmosphere with 5% CO_2_ at 37 °C and 95% relative humidity. All experiments were performed on cells between passages 4 and 8. The biological activity of the three EVOO-Pes, DTIC, OCEIN, and OCAL, as well as biological activity of the three EVOO-PEs following 6 h pre-incubation with DTIC was determined using a CellTiter 96^®^AQueous One Solution Cell Proliferation Assay. In short, 100 µL of cell cultures was seeded into 96-well tissue culture plates (A375 and HaCaT at a concentration of 2 × 10^3^ cells/well, A375M at 7 × 10^3^ cells/well). The next day, the medium was aspirated and media containing different concentrations of compounds were added to each well. All test compounds except DTIC were initially dissolved in DMSO and serially diluted in culture medium to obtain the final concentrations. DTIC was initially dissolved in culture medium, warmed to 37 °C, activated during 1 h exposure to light, and diluted in culture medium. The controls contained the test model cells and culture medium (containing either the complete medium or the DMSO in the highest final concentration, 0.2% and 0.4%) but no test compounds. The cells were incubated for 48 h before biological activity was measured. When investigating the combined effect, we exposed cells to DTIC for 6 h, removed the medium, and exposed the cells to medium containing EVOO-PE extracts for the next 24 h. In addition to the non-treated cells, the controls for experiments with DTIC pre-incubation were cells that had been exposed to DTIC for 6 h and cultured in a complete medium afterwards (without EVOO-PE). After incubation, media were aspirated, and 90 µL of serum-free medium containing MTS was added, according to the manufacturer’s instructions. After 1.5 h of incubation in the dark, the absorbance was measured at a wavelength of 490 nm using a microplate reader, Victor2-1420 Multilabel Counter (PerkinElmer, Yokohama, Japan). A negative control (medium without cells) was used as a blank. All experiments were performed in hexaplicate.

### 3.8. Statistical Analysis

The GraphPad Prism software version 9.0.0 for Windows (GraphPad Software, San Diego, CA, USA) [84], was used for the determination of inhibitory concentration as well as for statistical analysis. The normality of the data distribution was accessed using Kolmogorov–Smirnov and Shapiro–Wilk tests. The IC_60_ and IC_50_ values (concentrations that reduced cell growth to 60% or 50% of the initial value, respectively) were determined using a nonlinear regression curve fit. Analysis of variance (ANOVA) tests were used to assess significant differences among treatments, as well as to assess significant differences between quantified analytes in different samples of EVOOs. A principal component analysis (PCA) was carried out on the biological activity data. Results are shown as the mean values ± standard deviation (SD), or, in the case of IC determination, mean values ± standard error of mean (SEM), as allowed by the software. All *p*-values less than 0.05 were considered statistically significant. The results were expressed as mean values ± standard deviation (SD).

## 4. Conclusions

To our knowledge, this is the first report on the effects of EVOO-PEs on the biological activity of melanoma cells, including both amelanotic cells (A375) and their metastatic variant (A375M), as well as non-tumorous keratinocytes HaCaT. The composition profiling of three different EVOOs from autochthonous cultivars from Croatia included the determination of the secoiridoids OCAL, OCEIN, oleuropein aglycone, ligstroside aglycone, oleokoronal, oleomissional, and *S*-(*E*)-elenolide using ^1^H qNMR spectroscopy, as well as HTyr, Tyr, pinoresinol, cinnamic acid, apigenin, total polyphenols, *o*-diphenols, and total flavonoids using HPLC-DAD and spectrophotometric analysis. The results showed the largest difference mostly in oleacein, oleuropein, ligstroside aglycone, and *S*-(*E*)-elenolide. The biological effect of EVOO-PEs was compared to the effect of pure OCEIN and OCAL and also investigated after the cells’ exposure to chemotherapeutic DTIC.

The results showed that lower concentrations of EVOO-PEs may be sufficient to achieve a considerable, but not complete, inhibitory effect only on amelanotic and not non-tumorous cells, even when the EVOO-PEs are administered alone, and that EVOO-PEs applied after DTIC can, to a moderate extent, enhance its effect. Our results indicate that extreme caution is needed when applying high concentrations of oils because, although they are toxic to non-metastatic amelanotic cells, the overall impact may be unfavourable since the viability of non-tumorous cells is drastically reduced, while metastatic cells are not affected. The observed effects seem to be related; however, they are not limited to the content of secoiridoids, such as OCEIN and OCAL. Further studies are needed to obtain better insights into the impact of particular substances and their interplay, as well as on the detailed mechanisms responsible for the cellular response to EVOOs.

Altogether, our results indicate that EVOOs, in addition to their numerous known beneficial effects, have a certain potential for skin cancer treatment, especially in terms of supportive therapy in melanoma treatment or the prevention of melanoma progress in the early stage, which can be more or less prominent depending on the EVOO composition profile. However, the fact that an adverse effect of EVOOs at high concentrations was observed in non-tumorous cells should not be overlooked and indicates that special attention should be paid even when using EVOO in preparative cosmetics.

## Figures and Tables

**Table 1:** Total polyphenols, *o*-diphenols, and flavonoids in EVOOs.

	EVOO (Cultivar)		
	Žižolera	Bjelica	Crnica
TP (mg GAE/kg EVOO ± SD)	379 ± 15 ^A^	423 ± 38 ^A^	344 ± 41 ^A^
*o*-diphenols (mg GAE/kg EVOO ± SD)	216 ± 9 ^A^	122 ± 11 ^B^	137 ± 6 ^B^
TF (mg CE/kg EVOO ± SD)	392 ± 10 ^A^	230 ± 42 ^B^	160 ± 4 ^B^

EVOO—extra virgin olive oil; TP—total polyphenols; GAE—gallic acid equivalent; TF—total flavonoids; CE—catechin equivalent; SD—standard deviation. The means within each row labelled by different capital letters (^A^ and ^B^) are significantly different (one-way ANOVA test, *p* ≤ 0.05).

**Table 2:** Phenolic compounds/secoiridoids in EVOOs determined by ^1^H qNMR and HPLC.

Phenolic Compounds/ Secoiridoids (mg/kg EVOO ± SD)	EVOO (Cultivar)		
	Žižolera	Bjelica	Crnica
Oleocanthal	123 ± 0.1 ^A^	215 ± 43 ^A^	157 ± 31 ^A^
Oleacein	216 ± 0.3 ^A, B^	125 ± 1 ^A^	329 ± 83 ^B^
Oleuropein aglycone	215 ± 1 ^A^	75 ± 2 ^B^	19.4 ± 0.9 ^C^
Ligstroside aglycone	30 ± 1 ^A^	50 ± 4 ^B^	7.7 ± 0.3 ^C^
Oleokoronal	113 ± 9 ^A^	152 ± 9 ^B^	94.3 ± 5.8 ^A^
Oleomissional	88 ± 2 ^A^	36 ± 1 ^B^	96.3 ± 7.8 ^A^
*S*-(*E*)-elenolide	1054 ± 20 ^A^	164 ± 14 ^B^	514 ± 32 ^C^
Hydroxytyrosol	5.95 ± 0.12 ^A^	4.85 ± 0.10 ^B^	1.38 ± 0.17 ^C^
Tyrosol	3.07 ± 0.08 ^A^	5.47 ± 0.42 ^B^	1.91 ± 0.13 ^C^
Cinnamic acid	0.90 ± 0.09 ^A^	0.41 ± 0.02 ^B^	0.59 ± 0.11 ^B^
Pinoresinol	13.3 ± 0.7 ^A^	5.40 ± 0.33 ^B^	6.05 ± 0.57 ^B^
Apigenin	2.53 ± 0.26 ^A^	0.87 ± 0.01 ^B^	2.61 ± 0.16 ^A^

EVOO—extra virgin olive oil; SD—standard deviation. The means within each row labelled by different capital letters (^A–C^) are significantly different (one-way ANOVA test, *p* ≤ 0.05).

**Table 3:** Biological activity of EVOO-PEs, OCEIN, OCAL, and DTIC in A375, A375M, and HaCaT cells, expressed as IC_60_ and IC_50_ (in % *v*/*v* of EVOO-PE ± SEM or µM ± SEM, respectively).

		EVOO-PE Cultivar			OCEIN	OCAL	DTIC
		Žižolera	Bjelica	Crnica			
Cell Line	IC	%*v*/*v* of EVOO-PE ± SEM			µM		
A375	60	0.036 ± 0.002	0.057 ± 0.002	0.037 ± 0.002	101.052 ± 4.610	58.488 ± 1.836	214.358 ± 13.449
	50	0.042 ± 0.002	0.067 ± 0.002	0.047 ± 0.002	112.933 ±4.925	67.475 ± 1.863	361.716 ± 15,301
A375M	60	0.355 ± 0.011	0.401 ± 0.020	0.491 ± 0.052	ND	ND	709.780 ± 99.634
	50	0.401 ± 0.012	0.505 ± 0.034	0.546 ± 0.083	ND	ND	872.150 ± 62.158
HaCaT	60	0.126 ± 0.004	0.113 ± 0.003	0.187 ±0.006	103.061 ± 2.086	112.827 ± 2.904	1137 ± 93.752
	50	0.152 ± 0.004	0.133 ± 0.003	0.233 ± 0.007	114.351 ± 2.303	130.473 ± 3.215	1636.615 ± 122.559

EVOO-PE—extra virgin olive oil phenolic extract, OCEIN—oleacein, OCAL—oleocanthal, DTIC—dacarbazine; A375 and A375M—human melanoma cell lines; HaCaT—spontaneously immortalized keratinocyte cell line; IC_60_ and IC_50_—the concentration required to decrease biological activity, determined by an MTS test, to 60% or 50%, respectively, calculated using GraphPad Prism 9 software; SEM—standard error of mean; ND—not possible to determine. Numbers in italics represent values determined by extrapolation, which should be interpreted with caution.

## Data Availability

Not applicable.

## Supplementary Materials

The following supporting information can be downloaded at: https://www.mdpi.com/article/10.3390/molecules27103310/s1, Figure S1. ^1^H NMR spectrum of the aldehydic region of EVOO-PEs. (a) Žižolera; (b) Bjelica.; Figure S2. Calibration curves of oleocanthal (a) and oleacein (b) used for ^1^H NMR quantitation. Figure S3. HPLC chromatogram of EVOO-PEs at 278 nm. (a) Žižolera; (b) Bjelica (Ref. [79] in Manuscript), (c) Crnica. 1: Hydroxytyrosol, 2: Tyrosol, 3: Pinoresinol, 4: Cinnamic acid, 5: Apigenin.; Figure S4. DMSO does not affect cell viability significantly.; Figure S5. Effect of various concentrations of oleacein (OCEIN) and oleocanthal (OCAL) on cellular metabolic activity.; Figure S6. Effect of various concentrations of dacarbazine (DTIC) on cellular metabolic activity.; Table S1. Spectrophotometric analysis of phenolic compounds in EVOOs.; Table S2. ^1^H NMR analysis of secoiridoids in EVOOs.; Table S3. HPLC-DAD analysis of phenolic compounds in EVOOs.

## Sample Availability

Samples of the compounds are not available from the authors.
