# Supplementary material for: Extra Virgin Olive Oil Secoiridoids Modulate the Metabolic Activity of Dacarbazine Pre-Treated and Treatment-Naive Melanoma Cells
Source: Molecules. 2022 May 21;27(10):3310. doi: 10.3390/molecules27103310 (PMC9146374; doi:10.3390/molecules27103310)
Supplement: Supplementary file 1 [file molecules-27-03310-s001.zip › molecules-1733338 - Supplementary materials Figure S1-S6 Revised.pdf]

Supplementary Materials

Article

Extra Virgin Olive Oil Secoiridoids Modulate Metabolic Activity of Dacarbazine Pre-treated and Treatment-naive Melanoma Cells

Azra Kugić[1], Sanja Dabelić [1], Cvijeta Jakobušić Brala [1], Nina Dabelić [2] and Monika Barbarić [1,*]

1 University of Zagreb Faculty of Pharmacy and Biochemistry, A. Kovačića 1; 10 000 Zagreb, Croatia; akugic@student.pharma.hr (A.K.); sanja.dabelic@pharma.unizg.hr (S.D.); cjakobus@pharma.unizg.hr (C.J.B.); monika.barbaric@pharma.unizg.hr (M.B.)

2 University Hospital Center Sestre Milosrdnice, Referral Center for Melanoma of Ministry of Health of Republic of Croatia, Department of Oncology and Nuclear Medicine, Vinogradska 29, 10 000 Zagreb, Croatia; nina.dabelic@kbcsm.hr (N.D.)

* Correspondence: monika.barbaric@pharma.unizg.hr; Tel.: +385-01-6394-472

Received: date; Accepted: date; Published: date

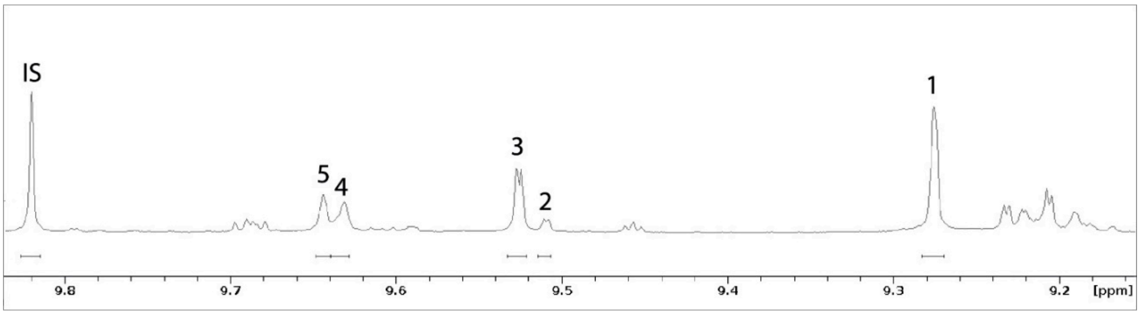

(**a**)

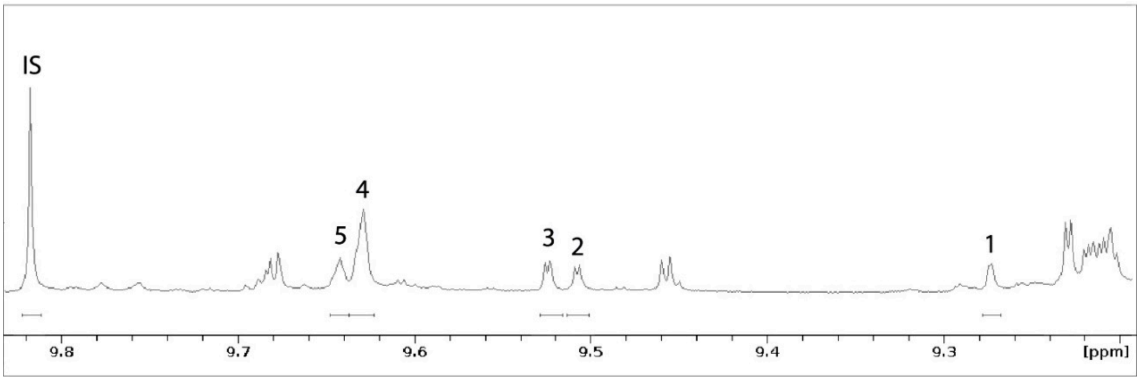

(**b**)

Figure S1. ^{1}H NMR spectrum of the aldehydic region of EVOO-PEs. (**a**) Žižolera; (**b**) Bjelica. 1: (*S*)-(*E*)-elenolide, 2: Ligstroside aglycone, 3: Oleuropein aglycone, 4: Oleocanthal, 5: Oleacein. IS – internal standard (syringaldehyde).

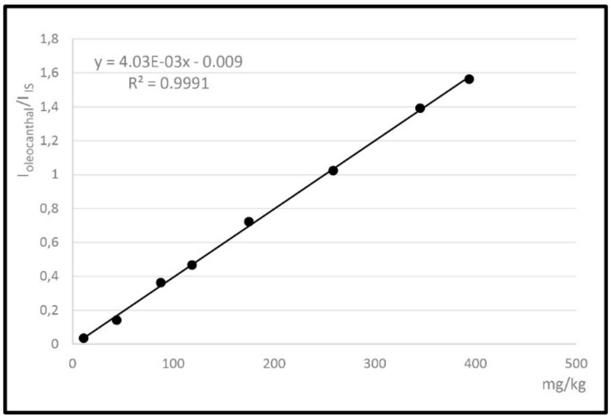

(**a**)

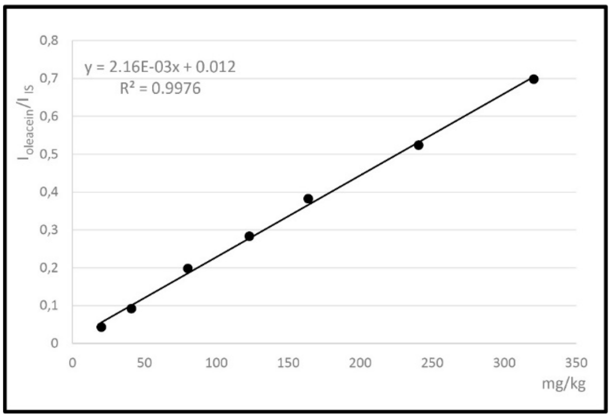

(**b**)

Figure S2. Calibration curves of oleocanthal (**a**) and oleacein (**b**) used for ^{1}H NMR quantitation (y = ax + b; y = peak area ratio, x = concentration of phenolic compound expressed in mg/kg). I – peak integration area. IS – internal standard.

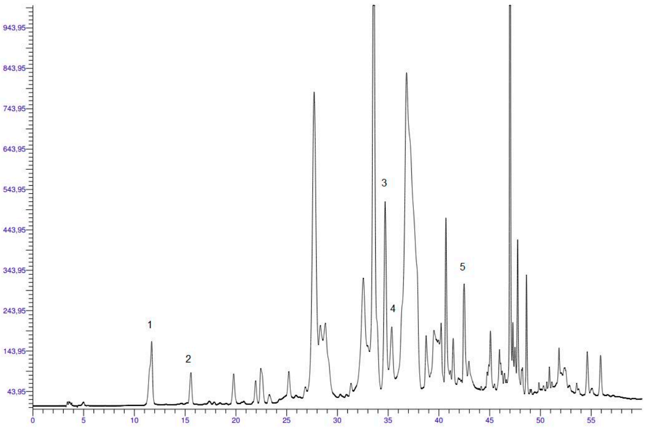

(**a**)

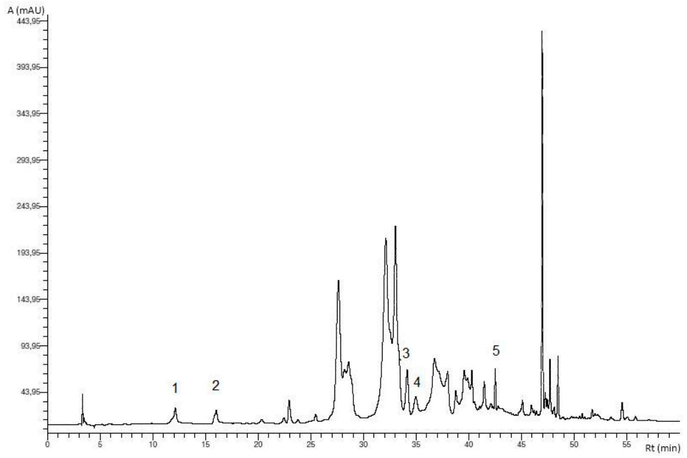

(**b**)

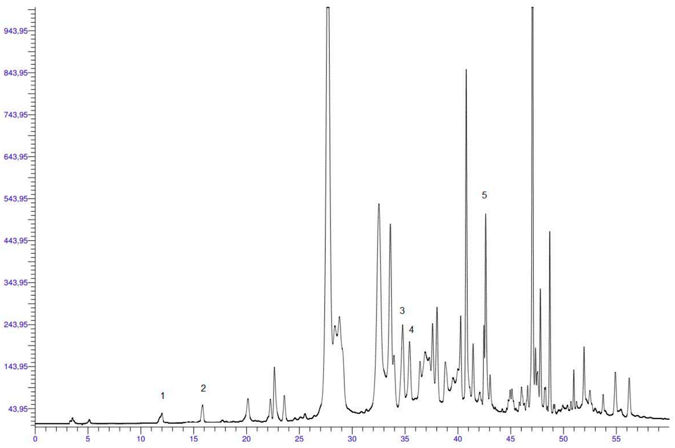

(**c**)

Figure S3. HPLC chromatogram of EVOO-PEs at 278 nm. (**a**) Žižolera; **(b**) Bjelica (Ref 79 in Manuscript); (**c**) Crnica. 1: Hydroxytyrosol, 2: Tyrosol, 3: Pinoresinol, 4: Cinnamic acid, 5: Apigenin.

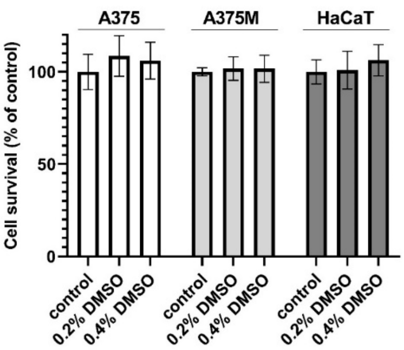

Figure S4. DMSO does not affect cell viability significantly. Three different cell lines (A375, A375M, and HaCaT) were exposed for 48 hours to DMSO in 0.2 and 0.4% concentrations. Metabolic activity was measured using MTS assay. The results of the six experiments, each performed in hexaplicate, are expressed as mean percentage of cell viability compared to non-treated cells ± SD. There were no statistically significant differences (p<0.05) compared to non-treated cells.

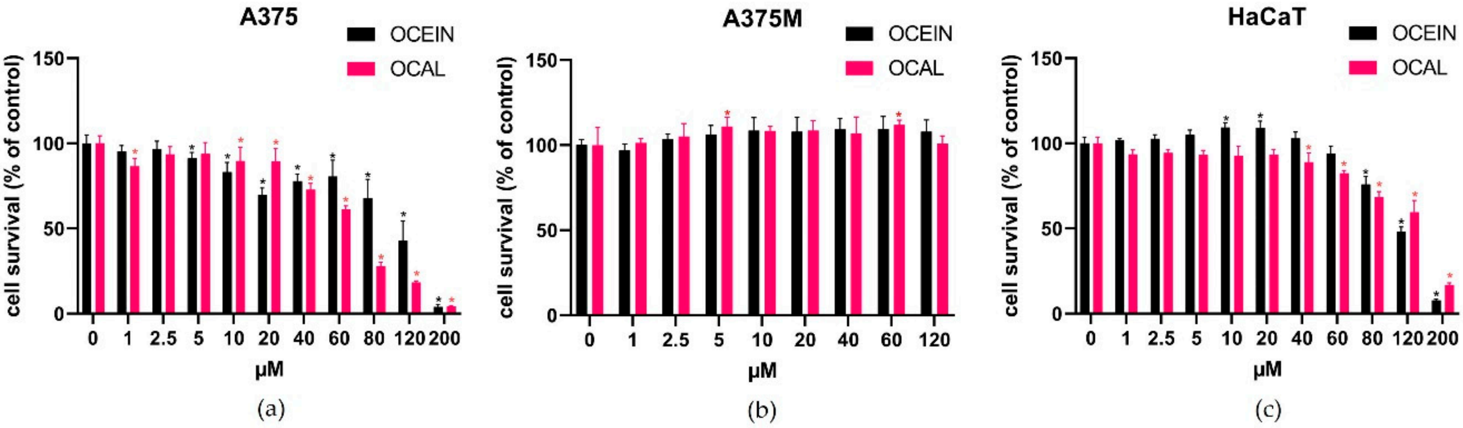

Figure S5. Effect of various concentrations of oleacein (OCEIN) and oleocanthal (OCAL) on cellular metabolic activity. Three cell lines: (**a**) A375 melanoma cell line, (**b**) A375M metastatic melanoma cell line, and (**c**) HaCaT immortalized keratinocytes; were exposed for 48 hours to various concentrations of OCEIN or OCAL. Metabolic activity was measured using MTS assay. Results of the six experiments are expressed as mean percentage of cell viability compared to non-treated cells ± SD. Statistically significant differences (p<0.05) compared to non-treated cells are marked with an asterisk.

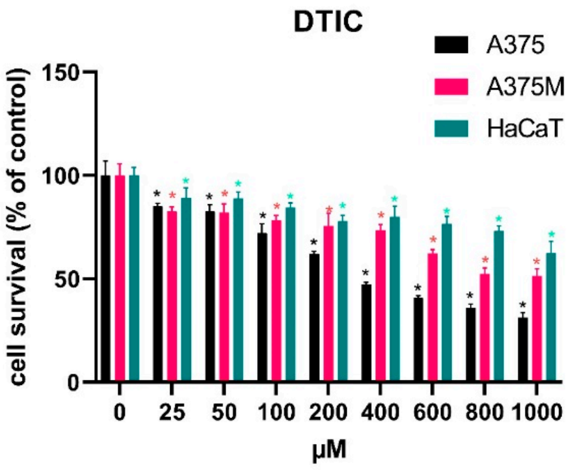

Figure S6. Effect of various concentrations of dacarbazine (DTIC) on cellular metabolic activity. Three cell lines: A375 melanoma cell line, A375M metastatic melanoma cell line, and HaCaT immortalized keratinocytes; were exposed for 48 hours to various concentrations of DTIC. Metabolic activity was measured using MTS assay. Results of the six experiments are expressed as mean percentage of cell viability compared to non-treated cells ± SD. Statistically significant differences (p<0.05) compared to non-treated cells are marked with an asterisk.
